# Wearable, Environmental, and Smartphone-Based Passive Sensing for Mental Health Monitoring

**DOI:** 10.3389/fdgth.2021.662811

**Published:** 2021-04-07

**Authors:** Mahsa Sheikh, M. Qassem, Panicos A. Kyriacou

**Affiliations:** Research Centre for Biomedical Engineering, School of Mathematics, Computer Science & Engineering, City, University of London, London, United Kingdom

**Keywords:** mental health monitoring, wearables, personal sensing, physiological and behavioral monitoring, digital phenotyping

## Abstract

Collecting and analyzing data from sensors embedded in the context of daily life has been widely employed for the monitoring of mental health. Variations in parameters such as movement, sleep duration, heart rate, electrocardiogram, skin temperature, etc., are often associated with psychiatric disorders. Namely, accelerometer data, microphone, and call logs can be utilized to identify voice features and social activities indicative of depressive symptoms, and physiological factors such as heart rate and skin conductance can be used to detect stress and anxiety disorders. Therefore, a wide range of devices comprising a variety of sensors have been developed to capture these physiological and behavioral data and translate them into phenotypes and states related to mental health. Such systems aim to identify behaviors that are the consequence of an underlying physiological alteration, and hence, the raw sensor data are captured and converted into features that are used to define behavioral markers, often through machine learning. However, due to the complexity of passive data, these relationships are not simple and need to be well-established. Furthermore, parameters such as intrapersonal and interpersonal differences need to be considered when interpreting the data. Altogether, combining practical mobile and wearable systems with the right data analysis algorithms can provide a useful tool for the monitoring and management of mental disorders. The current review aims to comprehensively present and critically discuss all available smartphone-based, wearable, and environmental sensors for detecting such parameters in relation to the treatment and/or management of the most common mental health conditions.

## Introduction

Mental illness is a health condition that alters a person's thoughts, feelings, and/or behaviors and causes the person distress and difficulty in functioning. The aggregate lifetime prevalence of common mental disorders across 39 countries has been estimated at around 30% (1). Mental health disorders are a major contributor to global disease burden due to their high prevalence, impairment of critical brain functions, and clinical course that is either chronic or remitting and relapsing. Some of the general symptoms and warning signs for mental disorders include marked personality change, inability to cope with problems and daily activities, strange and grandiose ideas, extreme mood swings, excessive anxieties, violent behavior, and thinking or talking about suicide or harming oneself (2).

Conventionally, mental disorders are diagnosed by self-report screening questionnaires or based on the *Diagnostic and Statistical Manual of Mental Disorders V*. Therefore, clinical diagnosis of mental disorders is achieved through the patient's subjective description of the symptoms, interviews and psychological questionnaires, and the physician's own expertise. However, the conventional assessment of psychiatric disorders based on a patient's or informant's recall is subject to inherent biases, and unreliability as the key feature in mental disorders is the variation of mood over time. In addition, mental health disorders are often chronic and relapsing in nature, and thus, long-term treatment management and assessment are essential for patient symptom reduction and recovery, but this is difficult to achieve with traditional methods, which rely on retrospective reports that are subject to recall bias. The lack of accurate methods for characterizing behavior and the need for regular monitoring of mental health conditions poses several physical and economical challenges, which, in recent years, has prompted great interest in digital phenotyping of mental health disorders through monitoring of physiological and behavioral parameters that can be translated into biomarkers of conditions, such as depression, anxiety disorders, bipolar disorder, and schizophrenia.

Physiological measures, such as heart rate variability (HRV), skin temperature (ST), electromyography (EMG), blood volume pulse (BVP), blood pressure, and cortisol levels as well as behavioral data, including sleep duration, social activities, and voice features, are among the many factors influenced by many mental health disorders. Consequently, capturing physiological and behavioral data has been widely implemented for the monitoring and general management of mental health.

In general, context sensing, personal sensing, mobile sensing, and digital phenotyping are all terms referring to identifying behaviors that relate to physical and mental health (2). Personal sensing involves collecting and analyzing data from sensors embedded in the context of daily life to detect and measure physical properties. To translate the raw sensor data into markers of behaviors and states related to mental health, the captured data is converted into features that are used to define behavioral markers through machine learning (3). Smartphones and connected devices, such as smartwatches, can be used to monitor behavior through passive detection of location, acceleration, social activities, and voice features.

Passive collection of physiological and behavioral data has been implemented for the diagnosis of various mental health disorders. This includes a wide range of studies that have investigated smartphone-based, wearable, and environmental sensors for digital phenotyping and monitoring of mental health. Until recent years, direct measurement of physiological data (e.g., HR, sleep quality, skin conductance) has been impractical as such devices have traditionally been bulky, large, and expensive (4). However, the development of various wearable devices, such as wristbands, smartwatches, and fitness tracking devices, and the common use of smartphones have facilitated the collection of biologically significant data. As a result, new technologies are rapidly being developed in fostering mental health and will allow for collection and analysis of data derived from self-reports, monitoring of behavioral patterns, and physiological sensing. More importantly, patients with serious mental illnesses have mostly shown good adherence and interest in using such devices to collect physiological and behavioral data (4), which would provide a vital complement to clinical visits.

The enthusiastic consumer and patient use of mobile devices, the availability of high-speed wireless internet, and a high prevalence of connected mobile devices with operating systems have culminated in patient and provider interest in monitoring mental symptoms and capturing behavioral and physiological data. This paper provides a review on recent developments in digital phenotyping based on physiological and behavioral monitoring of symptoms related to mental health and discusses the impact of such technologies in mental health care as, despite the expanded interest in mobile and connected technology in the arena of mental health, very few e-tools have been well-studied and validated for their impact when implemented on a broad scale (4).

## Methods

**Figure 1:**
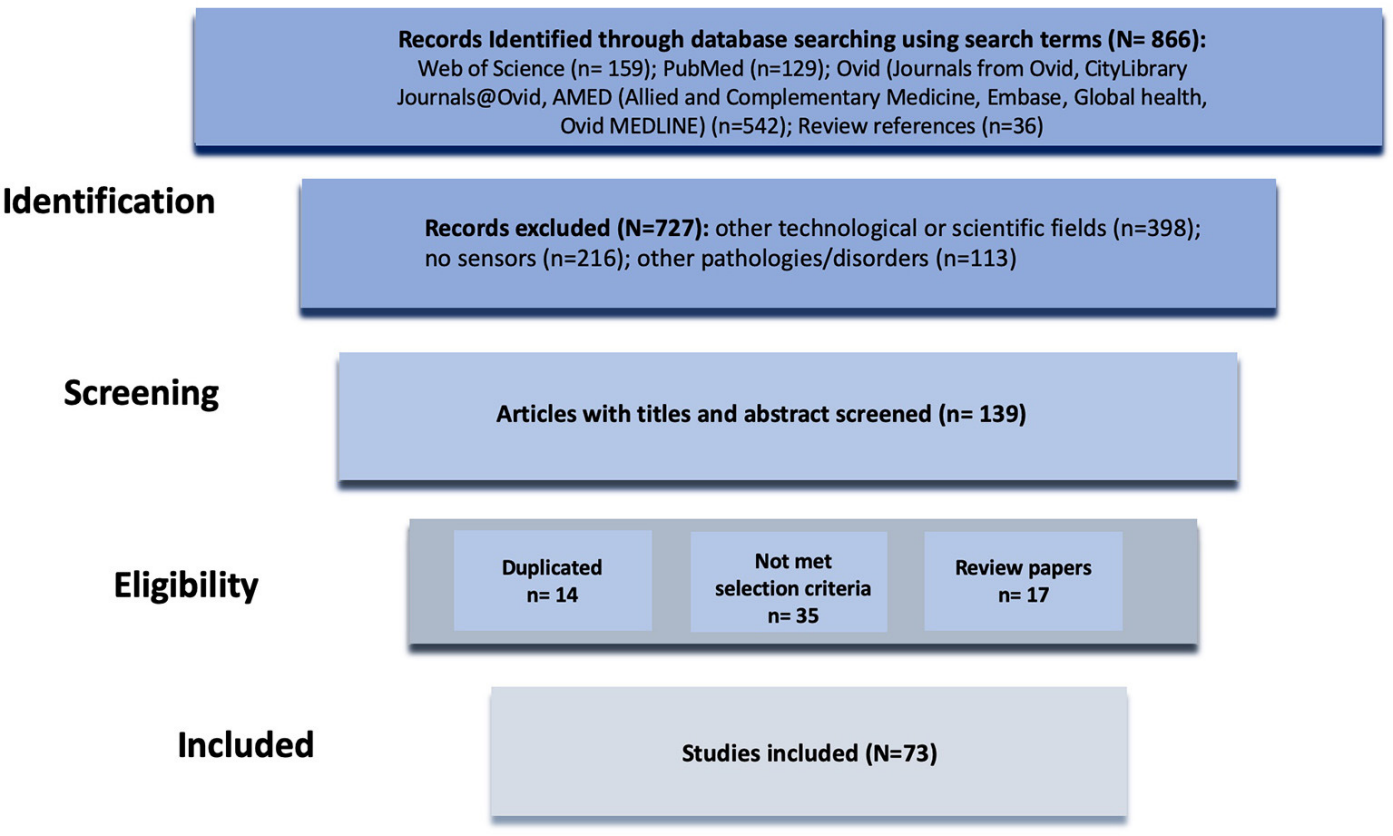
Diagram of the methodology used for the literature review process.

Searches of the literature were conducted in Web of Science, PubMed, and Ovid [including Journals from Ovid, CityLibrary Journals@Ovid, AMED (Allied and Complementary Medicine), Embase, Global health, and Ovid MEDLINE]. Keywords used in this search included “sensors,” “mental health monitoring,” “personal sensing,” “mental disorders,” “physiological and behavioral monitoring,” and “digital phenotyping.” Database searches yielded 851 results of which 21 were review papers. The references of relevant review papers were scanned to identify applicable studies. From the combinations of the keywords and 36 relevant articles found in review references, 866 articles were identified. Studies investigating physiological and behavioral monitoring in any condition other than mental disorders were excluded. Studies in which no sensing device was employed for monitoring physiological and behavioral parameters were also excluded. From careful analysis of titles and abstracts, 139 articles were identified, 73 of which met the inclusion criteria and were included for analysis (Figure 1).

**Figure 2:**
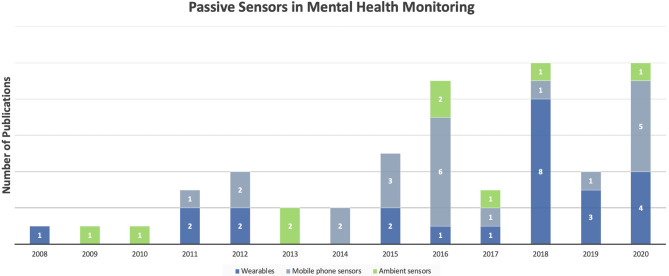
Number of publications investigating monitoring systems in mental health from 2008 to 2020.

The literature search yielded 73 studies, 55 of which investigated wearable, mobile phone, and ambient sensors, summarized in Tables 1–**3**. The remaining 18 papers focused on identifying behavioral and physiological changes linked to mental health and used existing monitoring systems without considering and investigating a specific sensing mode. These studies focused on identifying mental health–related physiological and behavioral changes, some of which are specifically discussed in section Mental Health–Related Physiological and Behavioral Changes. The years of publications analyzing different sensing devices for mental health monitoring ranged from 2008 to 2020 with the years 2015 to 2020 producing the majority of publications (Figure 2). Wearables and sensor-enabled smartphone applications constituted the majority of studies with 24 and 22 publications, respectively. A total number of nine studies were found to investigate ambient sensors in mental health monitoring.

**Table 1:** Wearable physiological and emotional monitoring research prototypes.

**Reference/product**	**Purpose**	**Device form factor**	**Sensors/parameters**	**Clinical application**
**Wearables**				
Jiang et al. (5), Newcastle University, UK, 2019	Audio sensing, motion detection, behavior monitoring	Watch	Light and temperature sensors, microphone, accelerometers	Anxiety, autism
Guo et al. (6), Purdue University, USA, 2016	Emotion recognition	Scarf	Heart rate sensor, Electrodermal Activity (EDA) sensor	Emotion regulation
Hui et al. (7), The University of Reading, 2018	Emotion recognition	Glove	PPG, electrodermal activity, skin temperature, and electromyography (EMG) sensors	Emotion regulation
MyFeel (8), Sentio Solutions Inc	Anxiety detection	wristband	Heart rate, electrodermal activity, and skin temperature	Emotion recognition and stress management
Reveal (9), Awake labs	Anxiety detection	Smartwatch	Heart rate, electrodermal activity, and skin temperature	Emotion recognition and stress management
Thync (10), Thync Global Inc	Increase energy and lower stress	Patch	Electrical Nerve Stimulation (TENS) and Transcranial Direct Current Stimulation (tDCS)	Neurostimulation
AutoSense (11), Washington, USA, 2011	Stress detection	Sensor Suite	Electrocardiogram (ECG) measurement, respiratory inductive plethysmograph (RIP), Galvanic skin response (GSR), skin and ambient temperature sensors, three-axis accelerometer	Stress management
Mobile Sensing Platform (MSP) (12), Choudhury et al., 2008	Automatic activity recognition	Wearable device	Microphone, visible light, pho-transistor, three-axis digital accelerometer, digital barometer, temperature, and digital compass	Cognitive assistance
Fletcher et al. (13), Boston, USA, 2011	ECG heart monitoring	Neoprene band	Electrodermal activity (EDA), 3-axis motion, temperature and electrocardiogram (ECG)	Posttraumatic stress disorder (PTSD), drug-addiction
Vidal et al. (14), Lancaster University, UK, 2011	Monitoring the link between eye movements and cognition	Wearable eye tracking equipment	Electrooculography (EOG)	Mental health monitoring
Psyche (15), Lanata et al., University of Pisa, Italy, 2015	Monitoring of pathological mood states in bipolar disorder	T-shirt	Electrocardiogram-Heart Rate Variability (HRV), piezoresistive sensors, tri-axial accelerometers	Bipolar disorder monitoring
Prociow et al. (16), University of Nottingham, UK, 2012	Monitoring of bipolar symptoms	Wearable and environmental sensors	Belt-worn accelerometer, wearable light sensor, and Bluetooth encounters, PIR motion sensors, door switches, remote control usage monitor	Bipolar disorder monitoring
Jin et al. (17), Newcastle, UK, 2020	Analyzing behavior signals and speech under different emotions	Wristband	6D acceleration and angular sensor, temperature and humidity sensor, MEMS microphones, audio code unit	Anxiety and depression monitoring
Can et al. (18), Istanbul,Turkey, 2019	Continuous stress detection	Samsung Smartwatches, Empatica E4 wristbands	Heart rate activity, skin conductance, and accelerometer signals	Stress detection
Dagdanpurev et al. (19), Tokyo, Japan, 2018	Measuring heart rate (HR), high frequency (HF) component of heart rate variability (HRV), and the low frequency (LF)/HF ratio of HRV before, during, and after the mental task	Fingertip sensor	Photoplethysmograph (PPG) sensor	Major depressive disorder (MDD) screening system
Horiuchi et al. (20), Kanagawa, Japan, 2018	Measuring frequency, duration, and velocity of eye blinks as fatigue indices	Smart glass	Optical sensor: dye-sensitized photovoltaic cells	Fatigue assessment
Minguillon et al. (21), Granada, Spain, 2018	Classifying stress levels based on EEG, ECG, EMG, and GSR	Portable system	RABio w8 (real-time acquisition of biosignals, wireless, eight channels) system, electroencephalography, electrocardiography, electromyography, and galvanic skin response	Stress detection
Tsanas et al. (22), Edinburgh, United Kingdom, 2020	Monitoring activity, sleep, and circadian rhythm patterns	Wrist-worn sensor	Actigraphy, light, and temperature data	Posttraumatic stress disorder (PTSD) monitoring
Razjouyan et al. (23), Houston, USA, 2020	Quantifying physical activity patterns and nocturnal sleep using accelerometer data	Chest-worn sensor	Accelerometer data	Identification of cognitive impairment
McGinnis et al. (24), Burlington, USA, 2018	Detection of internalizing disorders in children	Belt-worn measurement unit	Motion data	Early detection of depression and anxiety disorders
Correia et al. (25), Braga, Portugal, 2020	Monitoring changes in Heart rate variability (HRV) elicited by a mentally stressful task	Earlobe PPG sensor	PPG sensor	Stress detection
Mohino-Herranz et al. (26), Madrid, Spain, 2015	Detection of stress, mental overload and emotional status in real time	Vest	Electrocardiogram (ECG) and thoracic electrical bioimpedance (TEB) signals	Stress detection

**Figure 3:**
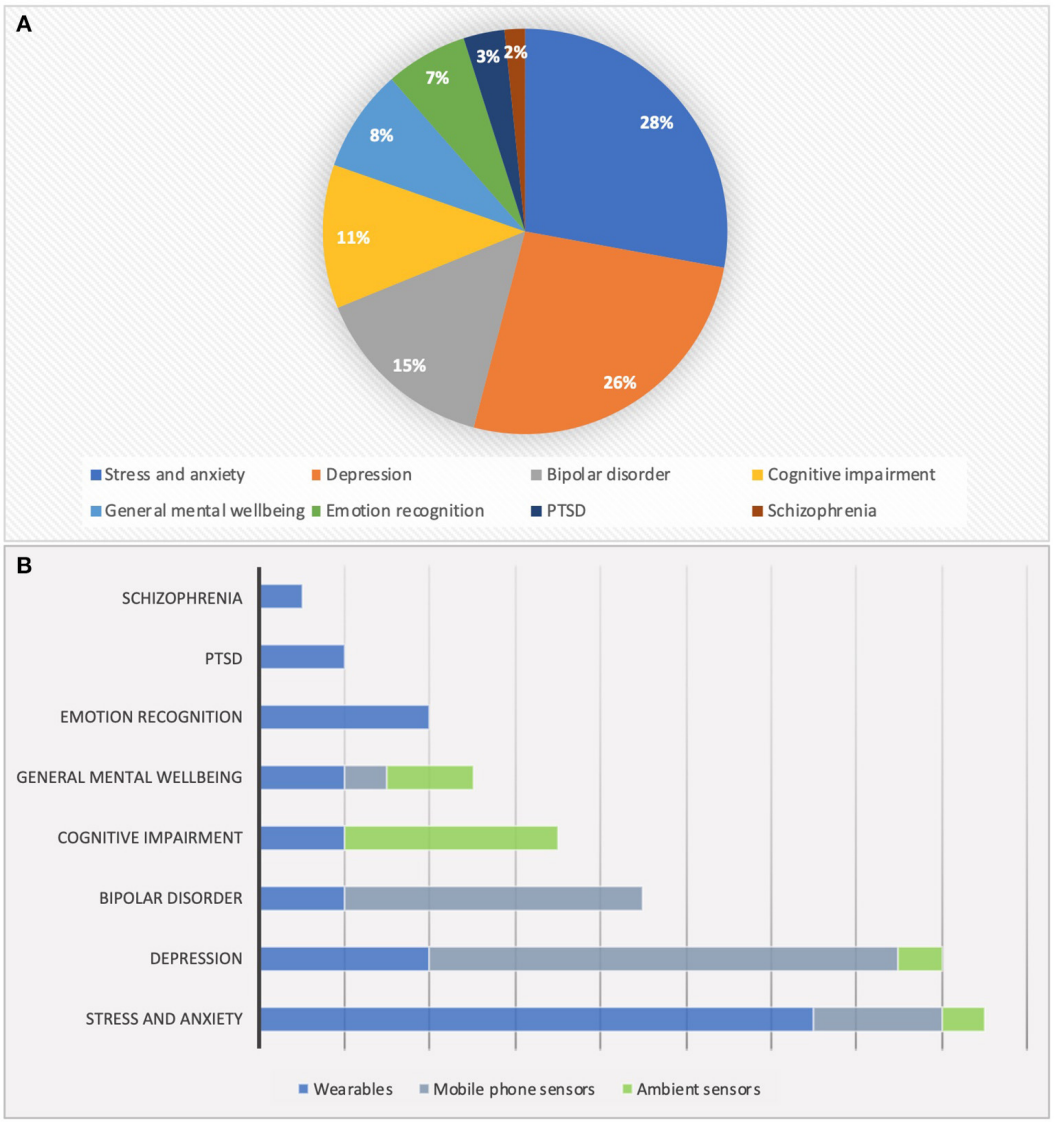
**(A)** Domain of the reviewed papers. **(B)** Investigated sensing devices for different mental disorders.

In sorting the included studies by application types, it is demonstrated that the majority of publications considered in this review investigated the use of monitoring systems for stress along with anxiety disorders and depression with just 2% difference between the two. Bipolar disorder monitoring, accounting for 15% of the studies, is the next well-studied condition for employment of behavioral and physiological monitoring systems. Schizophrenia and posttraumatic stress disorder (PTSD) monitoring comprise the smallest number of publications (Figure 3A). The application of different devices, including wearable, mobile phone, and ambient sensors, was also analyzed for each mental health condition. In the management of stress and anxiety disorders, wearable sensors were the most employed monitoring systems, mostly sensing physiological parameters, such as HR, electrodermal activity, and ST to evaluate anxiety levels. Whereas, for depression and bipolar disorder most of the studies used smartphone sensors as the monitoring system. In the monitoring of depression, the majority of studies focused on behavioral parameters, including physical activity, sleep duration, and conversation frequency to identify depressed mood. Furthermore, to assess the pathological mood states involved in bipolar disorder, physiological signals, such as HR and respiration activity, as well as behavioral parameters, such as mobility, social activity, sleep patterns, and voice features, were monitored. Although ambient sensors are generally the least investigated devices for mental health monitoring, they have been widely considered for detecting cognitive impairment (Figure 3B).

## Mental Health–Related Physiological and Behavioral Changes

A wide range of studies have demonstrated the association between physiological and behavioral patterns and different mental disorders. For example, it has been suggested that demonstrations of more phone activity from a combination of increased GPS position changes, erratic accelerometer movements, and increased social activity may be indicative of the manic phase in bipolar patients (55). Moreover, analysis of voice data through machine learning and natural language processing and detection of the pitch, tempo, and loudness of voice might serve as a potential marker of many psychiatric illnesses, such as depression, anxiety, and even suicidality (56). Portable noise sensors and GPS trackers have been used to identify links between mental health and personal noise exposure with high exposure to noise at the entire day level associated with worse mental health (57). Moreover, HRV, respiratory sinus arrythmia (RSA), electrodermal activity (EDA), ST, EMG, BVP, blood pressure, and cortisol levels are among the physiological markers that react to emotional experiences and can be used to detect stress and other emotional states (58). Wearables can also measure variables such as skin conductance and HR with greater asymmetries in skin conductance amplitude on the two sides of the body identified as an indicator of emotional arousal (3). Therefore, sensor data can be used to monitor human emotion states. However, the signals identified as physiological responses during various emotional states should be stable and reliable (59). Data from accelerometer-based wearable devices have also been used to detect the association between physical activity and depression (60). Although decreased levels of mobility and social communications were found to be indicative of higher depressive symptoms, factors such as light intensity and smartphone screen usage were unlikely to be predictive of depressed mood (61). Also, physiological sensing, such as measurement of HRV data as a marker of decompensation, has shown promising results in monitoring schizophrenia. Furthermore, Depp et al. suggests that, in patients with schizophrenia, less GPS mobility is associated with greater negative symptom severity and motivational deficit (62). However, it is important to note that these relationships are not simple, and due to their complexities, passive data may still have limited clinical utility (63). Moreover, the validity of the data collected with consumer technologies and how the information collected on these platforms correlates with the disorders should be further investigated (4).

## General Workflow of Mental Health Monitoring Systems

**Figure 4:**
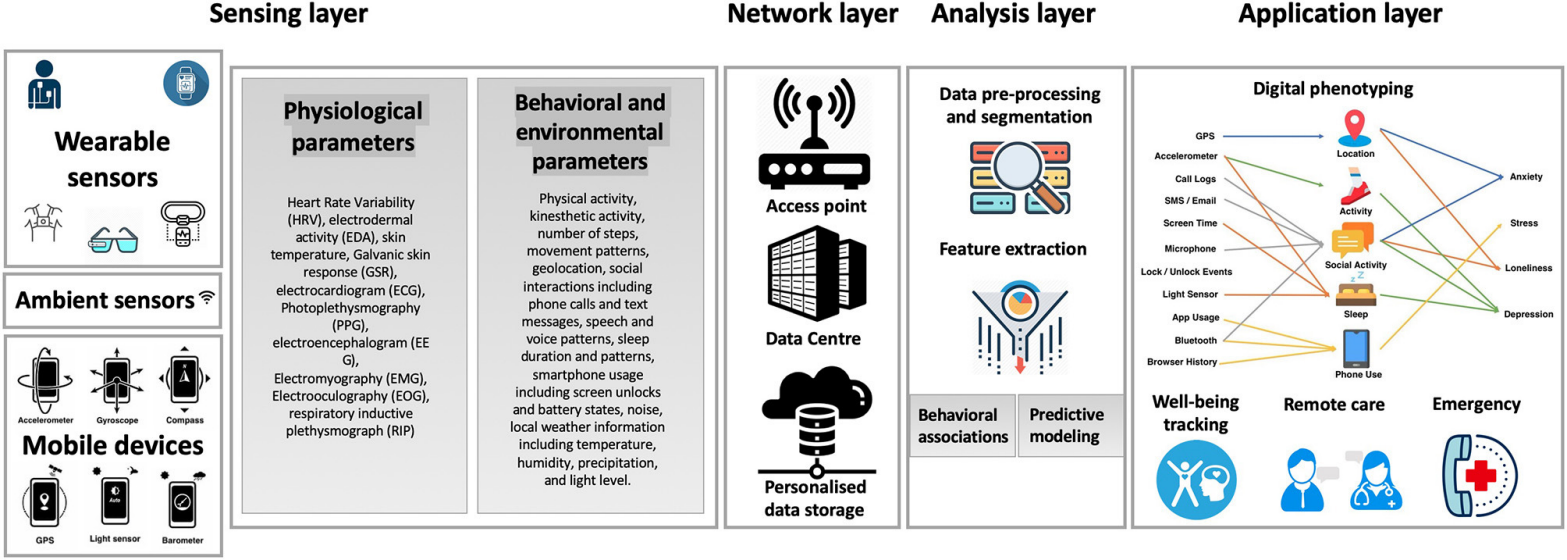
Overview of the workflow of mental health monitoring systems.

The internet of things–based layers involved in the structure of mental health monitoring systems is demonstrated in Figure 4. The general system collects health information from different sensing devices, including wearables, mobile phone sensors, and ambient sensors. These devices are capable of recording various physiological and behavioral data, including HR, lung function, sleep duration, etc. The sensing layer is typically followed by network and analysis layers, which involve employing the appropriate data acquisition and analysis techniques to eventually translate the raw sensor to achieve digital phenotyping in the application layer. The network layer comprising wired or wireless networks (e.g., Bluetooth, Wi-Fi) performs the collection and storage of data. Thereafter, the raw data is processed to extract features in the analysis layer, which is discussed in detail below. The activity classification step eventually categorizes the extracted features into different conditions in the application layer.

### Sensing Layer

An important aspect in the development of mental health monitoring systems is the sensing layer, whose role is to collect and transmit physiological, behavioral, and/or environmental parameters, thereby detecting changes in these in a continuous manner. To achieve the measurement of attributes related to individuals, various sensors, such as inertial sensors (e.g., accelerometer, gyroscope, or barometric pressure sensors) or physiological sensors (e.g., spirometer, ST, and blood pressure sensors) can be used. Moreover, a number of on-object sensors, such as environmental sensors, can be used for measuring indoor environmental conditions, including humidity and temperature.

The studies investigating wearable and mobile monitoring systems require constant interactions between fields such as medicine and computer science. Therefore, prerequisites are an important phase in these studies, some of which include the need for user consent on data collection; ethical approvals; and IT infrastructure, software, and hardware requirements (64). Furthermore, the type and number of sensors are often determined on the basis of application, all while considering their impact on factors such as battery consumption, obtrusiveness, and privacy (65). Sensor sampling rates, defined as the frequency at which the data is collected, should also be determined. Although high sampling rates provide more fine-grained pattern details, its impact on the device battery life is an important consideration (65). The studies are often performed as longitudinal and quantitative to provide the data required for building automatic predictive models and to measure a certain state over a period of time. Clinicians, health researchers, and patients should also couple mental health monitoring systems with frequent standard self-report scales, such as the Hamilton Depression Scale and the Young Mania Rating Scale, to evaluate their correlations. Therefore, the wearables and sensor-enabled smartphone applications are combined with self-reports to compare the sensor-derived measures with the reported conditions and determine the associations between the sensor-based auto-report and the self-report.

### Network Layer

The network layer is responsible for connecting all the devices in the sensory layer and allows the data to be collected, stored, transmitted, shared, and aggregated. To protect the participants' privacy, the data is anonymized, encrypted, transmitted via secure connections, and finally stored in secure servers. The latter can be performed in various ways; e.g., data acquired directly from smartphones can be stored in the internal device memory. Additional methods exist for data collected using wearables, such as watches and wristbands, with which data may be sent to a smartphone via Bluetooth or stored in secure storage platforms managing sensitive data (66) or to a remote server or a computer using wired, wireless, or internet connection. Various networks, including Blutooth, Wi-Fi, Zigbee, and cellular, can be adopted in the network layer.

### Analysis Layer

#### Data Labeling

Machine learning algorithms rely on training data to find patterns and generate predictive models. Therefore, the data labeling phase, including tagging the sensor data with their corresponding ground truth state, is important in training the final predictive models. The data labeling phase can happen on-site, at the hospital/clinic, or off-site while the participants perform their daily routines at home/work/etc. Data tagging can be achieved through periodic specialist/doctor assessments on-site or by phone (66, 67), using self-reports often presented by a mobile application in periodic intervals in which the participant is in charge of the labeling process (68, 69) or according to some event, such as labeling stress during an exam situation (70).

#### Data Analysis and Preprocessing

After collecting the data, exploratory data analysis and preprocessing are used to better understand and visualize the data and detect outliers. Preprocessing includes applying filters and transformations, such as scaling, quantization, binarization, and so on, to the raw data to reduce noise and remove outliers. Dimensionality reduction techniques, including principle component analysis (71) and multidimensional scaling (72), are among the most common preprocessing techniques. Feature extraction is then used to build feature vectors from the raw sensor data. Feature vectors are required for machine learning algorithms as they are representations of the original data. Arithmetic mean, standard deviation, min, max, skewness, kurtosis, root mean square, power spectrum density, energy, and correlation coefficient are some of the common extracted features for mental states detection (68, 73, 74).

#### Machine Learning Model Training

Different types of machine learning algorithms, including supervised, unsupervised, semi-supervised, transfer, and reinforcement learning, are used to find particular patterns and relationships from the vast amount of collected data and finally yield the appropriate predictive model(s). It should be noted that algorithms are often combined to get the final predictive models. However, there is a distinction between certain training schemes, such as user-dependent and user-independent (general) models. User-dependent models, trained with data from the specific user under consideration and capturing the specific behavior of each user, yield better results but require a lot of data training. User-independent models, trained with data from all other users excluding the target user, do not require any data for the target user but might not perform well for atypical users. Although Lu et al. suggest that user-dependent models perform much better for stress detection (75), some studies propose that hybrid models have the best of both user-dependent and -independent models (68, 76).

As mentioned previously, physiological and/or behavioral monitoring systems are often coupled with data analysis platforms whose role is to convert raw sensor data into measures of mental disorders. Accordingly, different machine learning software tools/libraries are used for evaluating and training personalized or general models. These include the Weka software and the scikit-learn library used for bipolar detection (77, 78) and anxiety recognition (79), respectively. Furthermore, InSTIL (Intelligent Sensing to Inform and Learn) is a software platform designed for digital phenotyping. The platform provides acquisition of sensor data from consumer smartphones and is being used by studies that seek to collect passive and active sensor signals (80). Another developed platform is the Remote Assessment of Disease and Relapse (RADAR)-base platform, which can be integrated with remote monitoring initiatives and caters to large-scale data collection (81). These platforms provide general remote data collection at scale, management of studies, and real-time visualizations and ensure user privacy and security. However, other tools, such as Matlab machine learning toolboxes, can be also used based on different application types.

### Application Layer

Remote mental health monitoring is of great significance to individuals with mental disorders as well as their caregivers and physicians. Physiological and behavioral patterns can reflect mental states of the patients, and thus, recording such data provides physicians and caregivers with a useful method for accurate intervention and diagnosis. For example, data from accelerometers, GPS, and mobile phone sensors can be used to detect physiological patterns associated with depression or depressed mood states involved in bipolar disorder. Furthermore, HR and EDA sensors can be employed to monitor physiological markers that react to emotional experiences and allow emotion recognition and the detection of stress levels. Sleep, mobility patterns, and conversation frequency and patterns are among the parameters used for monitoring schizophrenia. Last, microphone, visible light, accelerometer, temperature, and digital compass sensors are often utilized to provide cognitive assistance.

Mental health monitoring systems can be also used to facilitate monitoring of symptoms in home and hospital environments. Using these systems instead of the conventional questionnaires or manual tests delivers particular information for physicians and caregivers and, thus, potentially assists self-management of well-being, reduces health care costs, and avoids undesirable consequences in a personalized manner. Furthermore, employing these systems to provide ambient assisted living, particularly for the elderly, provides an emergency system that is essential for monitoring and detecting abnormal physiological and behavioral states.

## Applications of Physiological and Behavioral Monitoring Systems

A wide range of devices aimed at physiological monitoring are presented, including wristbands and watches, chest straps, vests, garments and shirts, patches and sleeves (82–87). A list of wearable physiological and emotional monitoring systems can be found in Table 1. Various mental health studies on personal sensing have also utilized mobile phone sensors as they are widely owned and contain a large number of embedded sensors able to detect behavioral markers, such as sleep, social context, mood, and stress (Table 2). The use of smartphone sensors has been investigated for detecting the presence and severity of several mental disorders, including depression, bipolar disorder, and schizophrenia (29, 31, 88–90). Additionally, environmental or ambient sensors are generally installed in a single room, a house, or an entire building to understand the context of that environment by gathering information from sensors and providing assistance to the inhabitants. Ambient sensors have been investigated in some mental health monitoring studies, especially for detecting cognitive impairment (Table 3). The sections below discuss in detail the various wearable, ambient, and smartphone-based systems that have been investigated in recent years for treatment and management of common mental health disorders.

**Table 2:** Smartphone-based physiological and emotional monitoring research prototypes.

**Reference/product**	**Purpose**	**Platform**	**Sensors/parameters**	**Clinical application**
**Mobile phone sensors**				
BeWell+ (27), Lin et al., USA, 2012	Evaluation of well-being based on physical activity, social interaction and sleep patterns	Sensor enabled smartphone application	Human voice, sleep, physical activity, and social interaction	Well-being monitoring
MoodRhythm, Matthews et al. (28), USA, 2016	Monitoring of bipolar symptoms	Sensor enabled smartphone application	Phone's light sensor, accelerometers, and microphone, as well as phone use events such as screen unlocks and battery charging state, communication patterns including SMS and call logs	Bipolar disorder monitoring
MONARCA (29), Faurholt-Jepsen et al., Copenhagen, Denmark, 2014	Monitoring of pathological mood states in bipolar disorder	Sensor enabled smartphone application	Phone usage, social activity measured as the number and length of incoming and outgoing phone calls and text messages, physical activity measured through step counter, mobility based on location estimation and speech activity collected by extraction of different voice features	Bipolar disorder monitoring
Evidence-Baes Behavior (eB2) app (30), Berrouiguet et al., Brest, France, 2018	Monitoring mobility patterns of patients with depression	Sensor enabled smartphone application	Physical activity, phone calls and message logs, app usage, nearby Bluetooth and Wi-Fi connections, and location	Depression monitoring
CrossCheck (31), Wang et al., New Hampshire, USA, 2016	Monitoring passive smartphone sensor data and self-reported indicators of schizophrenia	Sensor enabled smartphone application	Sleep, mobility, conversations, smartphone usage	Schizophrenia
SIMBA (32), Beiwinkel et al., Lüneburg, Germany, 2016	Tracking daily mood, physical activity, and social communication in bipolar patients	Sensor enabled smartphone application	Geolocation	Bipolar disorder monitoring
Ben-Zeev et al. (33), New Hampshire, USA, 2015	Analyzing stress, depression, and subjective loneliness over time	Smartphone sensor data	Speech, sleep duration, geospatial activity, and kinesthetic activity	Stress and depression monitoring
Jacobson et al. (34), Lebanon, USA, 2020	Predicting depressed mood within a day	Smartphone sensor data collected from “Mood Triggers” app	location, local weather information: temperature, humidity, precipitation, light level; heart rate information: average heart rate and heart rate variability; and outgoing phone calls	Depression monitoring
Pastor et al. (35), Barcelona, Spain, 2020	Digital phenotyping of patients with alcohol use disorder and anxiety symptoms	Smartphone sensor data collected from “HumanITcare” app	Sleep cycle, heart rate, movement patterns, and sociability	Monitoring anxiety symptoms and alcohol use disorder
Purple robot (36), Schueller et al., Chicago, USA, 2014	Depression detection	Sensor enabled web-based and smartphone application	physical activity, social activity	Depression monitoring
FINE, Dang et al., Hannover, Germany, 2016	Depression detection	Sensor enabled smartphone application	Smartphone use, social activity, movement	Depression monitoring
Mobilyze! (37), Burns et al., Chicago, USA, 2011	Prediction and intervention of depression	Sensor enabled smartphone application	Physical activity, social activity	Depression monitoring
PRIORI (38), Gideon et al., Michigan, USA, 2016	Analysis of mood in individuals with bipolar disorder	Sensor enabled smartphone application	Analysis of voice patterns collected from phone calls	Bipolar disorder monitoring
SIMPle 1.0 (39), Hidalgo-Mazzei et al., Barcelona, Spain, 2015	Bipolar disorder symptom management and psycho- educational Intervention	Sensor enabled smartphone application	Smartphone usage, calls, and physical activity	Bipolar disorder monitoring
Me app (40), Asare et al., Oulu, Finland, 2019	Predictive measures of major depressive disorder	Sensor enabled smartphone application	GPS location, physical activity, light, noise, screen interaction, battery, application notifications, calls, messages	Depression monitoring
SOLVD app (41), Cao et al., Houston, USA, 2020	Monitoring depressive symptoms of adolescents	Sensor enabled smartphone application	Steps, GPS, SMS, call, light, and screen time	Depression monitoring
Toi Meme app (42), Daregel et al., Paris, France, 2020	Monitoring of pathological mood states in bipolar disorder	Sensor enabled smartphone application	Motor activities (eg, number of steps, distance) measured using the smartphone's motion sensors	Bipolar disorder monitoring
DeMasi et al. (43), California, USA, 2017	Mood monitoring	Sensor enabled smartphone application	Activity and sleep tracking using accelerometer data	Depression and bipolar disorder monitoring
Mobile Sensing and Support (MOSS) app (44), Wahle et al., Zurich, Switzerland, 2016	Detecting depression and providing personalized CBT intervention	Sensor enabled smartphone application	WiFi, accelerometer, GPS, and phone use	Depression monitoring
Daus et al. (45), Stuttgart, Germany, 2020	Mood recognition in bipolar patients	Sensor enabled smartphone application	Location, Accelerometer, Smartphone usage, social interaction	Bipolar disorder monitoring

**Table 3:** Ambient mental health monitoring research prototypes.

**Reference**	**Sensors/parameters**	**Application**
Ramón-Fernández et al. (46), Alicante, Spain, 2018	Level of noise in the room, flow of people moving through it, temperature, luminosity, and air quality	Stress detection
Kim et al. (47), Michigan, USA, 2017	Passive infrared motion sensors	Depression monitoring
Dawadi et al. (48), Washington, USA, 2013	Motion sensors on the ceiling, door sensors on cabinets and doors, and item sensors on selected	Cognitive assessment
Alam et al. (49), Yongin, Korea, 2016	Web of objects system comprising a smart home environment and lightweight biosensors	Psychiatric emergency
Hayes et al. (50), Oregon, USA, 2009	Measures of walking speed and amount of activity in the home	Cognitive assessment
Mielke et al. (51), Braunschweig, Germany, 2020	Detection of Psychomotor agitation by collecting motion data	Mental health monitoring
Kasteren et al. (52), Amsterdam, Netherlands, 2010	Activity monitoring using wireless sensor systems	Cognitive assessment
Bradford et al. (53), Canberra, Australia, 2013	Sensor-based in-home monitoring system for the elderly	Cognitive assessment
Ribonia et al. (54), Cagliari, Italy, 2016	SmartFABER sensor network collecting behavioral anomalies in the elderly	Cognitive assessment

### Emotion and Autonomic Activity Recognition

**Figure 5:**
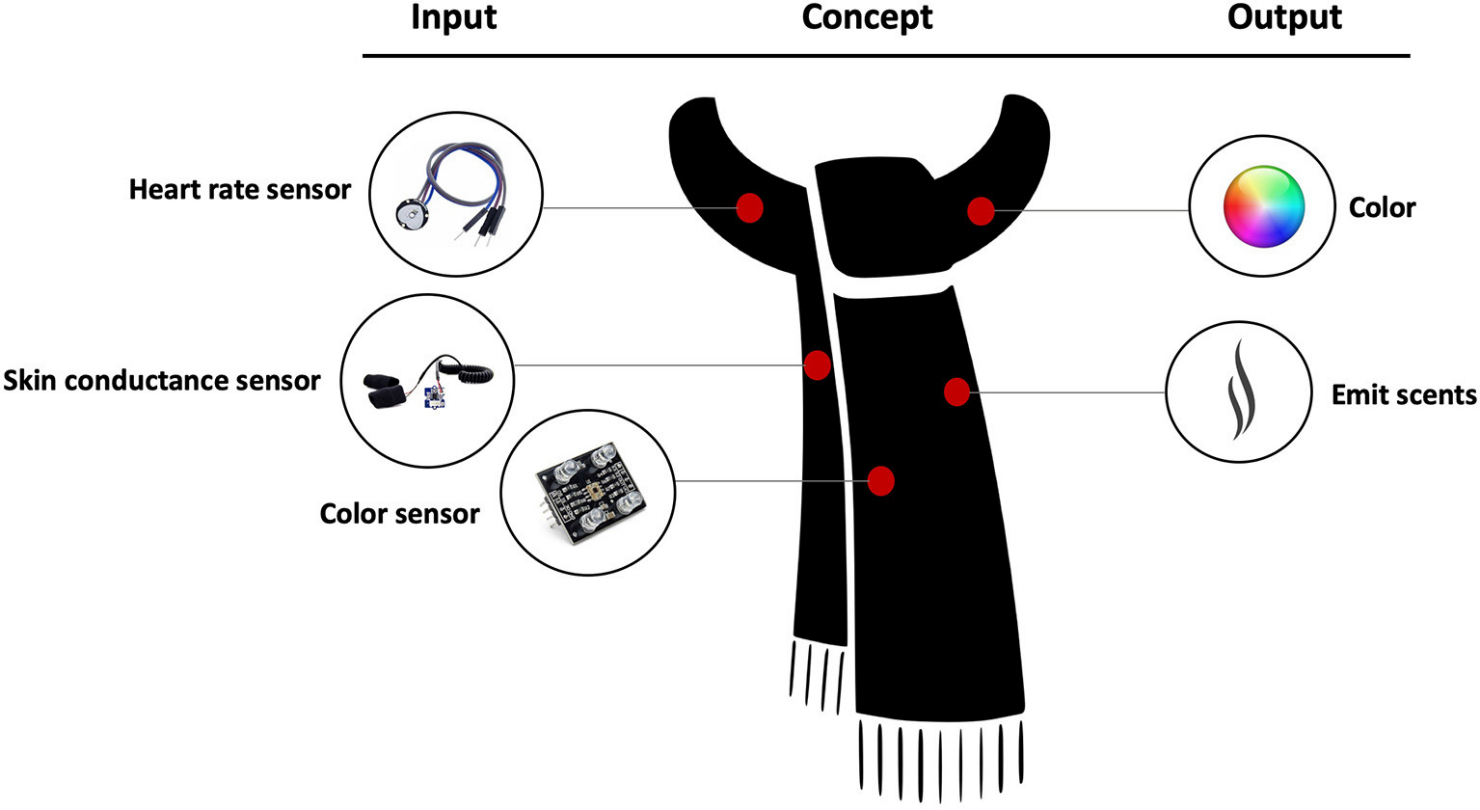
Concept of a smart scarf designed for emotion recognition (91).

Several wearable devices have been developed to monitor emotional information. The scarf-based monitor developed by Guo et al. uses an HR sensor and an EDA sensor to detect emotional states. The scarf also emits an odor and changes color to enhance the mood in response to negative emotions (Figure 5) (6). Similarly, a smart glove developed by Hui et al. utilizes a photoplethysmography (PPG) sensor for HR, EDA, ST, and EMG sensors to recognize different emotions, such as happiness, anger, fear, and disgust (7). To achieve emotion recognition using sensor data, a study by Jang et al. investigates the consistency on changes of bio-signals as physiological responses induced by six basic emotions—happiness, sadness, anger, fear, disgust, and surprise—using 60 different emotional stimuli. HR, skin conductance level (SCL), mean of ST (meanSKT), and mean of PPG (meanPPG) were measured before and during the presentation of the stimuli. The results suggest that biosensors are useful tools for emotion recognition as the physiological responses by six emotions were consistent over time; in particular, physiological features, such as SCL, HR, and PPG, are found to be very reliable (59). Moreover, a smart glass containing dye-sensitized photovoltaic cells, in which the optical sensors are positioned at the lateral side of the eye, has been designed for fatigue assessment based on eye blinks. The glass measures frequency, duration, and velocity of eye blinks as fatigue indices, which can potentially benefit the maintenance of physical and mental health (20). Also, a novel measurement technique using wearable eye tracking for mental health monitoring has been proposed based on the link between eye movements and cognition (14). A number of wearable monitoring systems have been studied to assess cognitive health in older adults. Using the data from a chest-worn sensor, it has been suggested that physical activity, including sedentary and light activities, percentage of walk and step count, and total sleep time and time in bed, are significant metrics for identifying cognitive frailty in older adults (23). Furthermore, a link between greater gait symmetry and better mental health has been identified as well by employing a high-speed 3-D motion sensing system to record gait mechanics in older adults (92). Another system is the mobile sensing platform, which is a small, wearable device designed for automatic activity recognition and cognitive assistance using on-body sensors, including microphone, visible light, pho-transistor, three-axis digital accelerometer, digital barometer, temperature, and digital compass (12).

**Figure 6:**
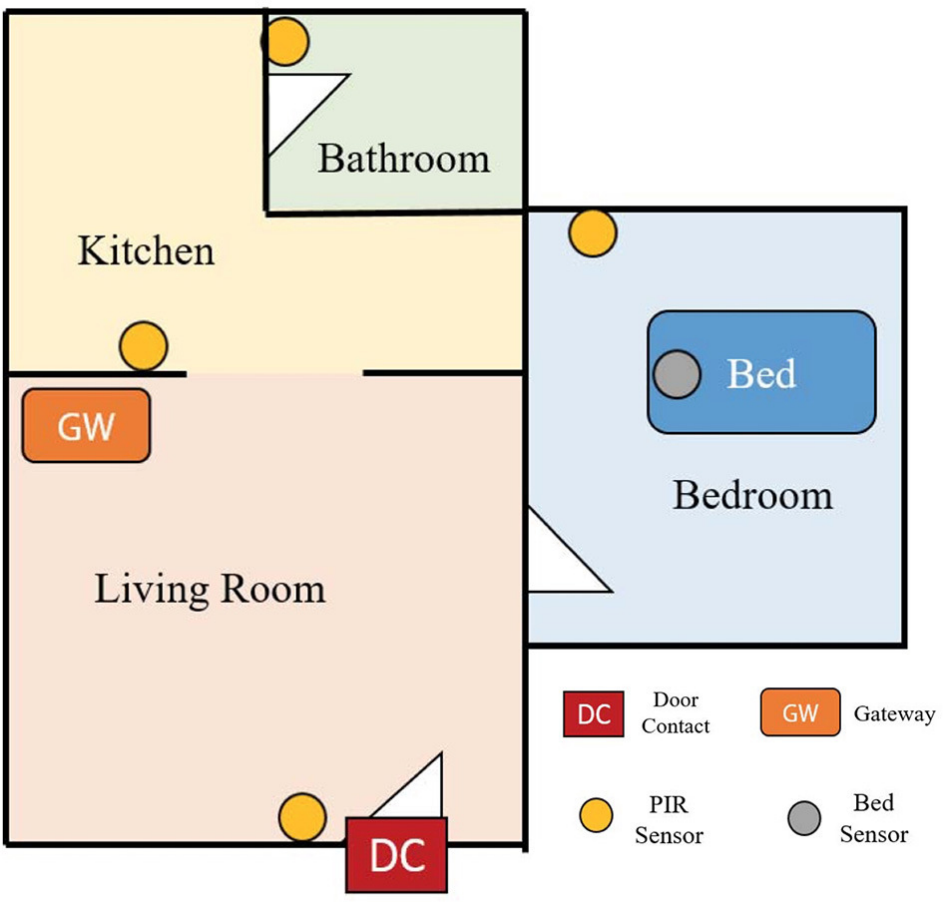
Sample configuration in an elderly house for long-term monitoring of depression. [Modified from Kim et al., *IEEE Sensors*, 2017 (47)].

Among studies employing ambient sensors, Alam et al. objectified different home appliances and sensors as a web of objects to create a smart home environment and combined it with lightweight biosensors and web-based psychiatric screening scales to assess patients' psychiatric symptoms (49). Additionally, with agitation being a symptom of many mental illnesses, including dementia, Mielke et al. attempted to detect psychomotor agitation patterns via installation of sensors in the apartment and building a smart home environment (51). The study proposes that the movement sequences identified as conspicuous or critical can be indicative of psychomotor agitation or possible mood and behavioral changes (51). Particularly, ambient sensors have been used in a couple of studies to achieve monitoring of cognitive health in older adults. In a study by Kasteren et al. state change sensors were located in doors, cupboards, refrigerator, etc., to provide an activity monitoring system and assist in caregiving for the elderly in their homes (52, 93). The Smarter Safer Homes project (53) and SmartFABER (54) sought to detect cognitive decline and abnormal behaviors in the elderly by creating sensor-based in-home monitoring systems that acquire data about the interaction of the senior with the home environment. Early detection of cognitive impairment has also been investigated in (50), by installing an unobtrusive activity assessment system containing motion sensors and contact sensors to monitor the activity of older adults in their homes. Moreover, passive infrared motion sensors have been employed to monitor the daily activities of elderly persons and achieve long-term depression monitoring by creating smart homes (Figure 6) (47). Last, a smart home test bed was designed at Washington State University represented by an apartment that was instrumented with motion sensors on the ceiling and sensors on cabinets and doors as well as sensors on selected kitchen items. The task quality of smart home activities were quantified to assess the cognitive health of the individuals (48).

### Stress and Anxiety Disorders

**Figure 7:**
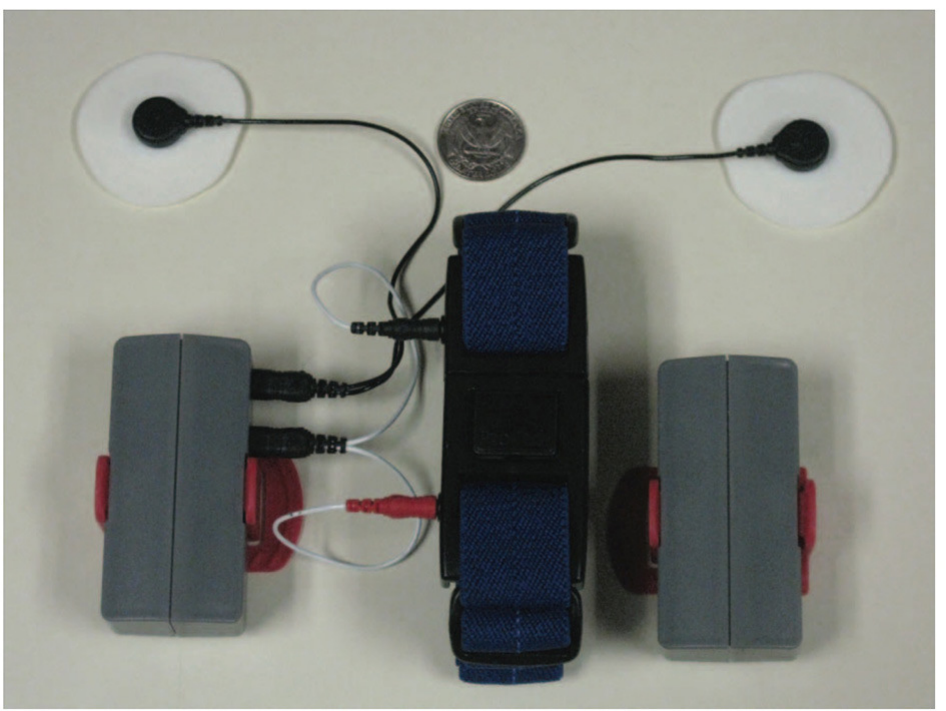
AutoSense sensor suite including ECG leads and RIP connectors plug in to the unit on the side. The red clip in the wearable packaging is used to attach the unit to the (blue) RIP band that goes around the chest [Modified from Ertin et al., *SenSys*, 2011(11)].

Anxiety disorders are characterized by unrealistic and excessive worry accompanied by symptoms such as extreme vigilance, motor tension, restlessness, muscle tension, and disturbed sleep (94). Various devices have been developed for emotion recognition and stress management applications. As such, the MyFeel wristband (8) developed by Sentio Solutions, Inc., and Reveal (9), collect HR, EDA, and ST to evaluate anxiety levels by applying data analytics and cognitive-behavioral techniques. Thync (10) developed by Thync Global, Inc., employs electrical nerve stimulation (TENS) and transcranial direct current stimulation (tDCS) as neuro-stimulation technologies to increase energy and lower stress. TENS delivers small electrical impulses that reach the brain through the spinal cord and may help alleviate stress levels by relaxing the muscles (95). tDCS has been found to prevent chronic stress in a preliminary animal study by modulating the neural activity through the application of a small current (96). A wireless sensor suite called AutoSense (11) also provides information relating to general stress with 90% accuracy by collecting and processing cardiovascular, respiratory, and thermoregularity measurements. AutoSense integrates six sensors in a small device and includes wireless transmission to a mobile phone in real time, which provides monitoring of physiological responses to real-life stressors and various behaviors that may be related to stress, such as drinking, smoking, physical activity, movement patterns, conversations, etc., (Figure 7) (11). Furthermore, in a study by Can et al., HR activity, skin conductance, and accelerometer signals have been captured by smart wearable devices to detect and discriminate stress during situations such as contests, higher cognitive load lectures, and relaxed time activities using machine learning methods. The study used Samsung Smartwatches and Empatica E4 wristbands for data acquisition with higher accuracy and data quality obtained with the later class of devices (18). The combination of different physiological parameters seems to provide the highest accuracy for emotion classification. For example, Can et al. suggest that a higher classification accuracy was obtained when heart activity is combined with EDA than when these modalities were used separately (18).

In relation to stress detection, a portable stress detection system based on the RABio w8 (real-time acquisition of biosignals, wireless, eight channels) system has been designed, which is combined with software that comprises an application programming interface and a graphical user interface. By inducing different levels of stress, the system is demonstrated to be efficient in classifying three levels of stress (stress, relaxed, and neutral). The team is also working on a more wearable version of this system as a cap embedded with all the electronics (21). Furthermore, Jiang et al. develop a long-term wearable well-being sensing watch that continuously and unobtrusively detects details of behaviors that might be related to the onset of anxiety and autism (5). The portable wearable device consists of audio feature calculation without preserving the raw data and a variety of digital sensors for collecting multimodal data from the environment as well as physiological signals and behavioral activity. It has been demonstrated that the social audio features captured by the device are highly correlated with questionnaire scores for monitoring individuals with high to low autism traits (5). An undergoing study is also aiming to develop digital phenotyping of patients with alcohol use disorder and anxiety symptoms using data collected from a smartphone and a wearable sensor. In this study, factors including sleep cycle, HR, movement patterns, and sociability are sent and saved to the HumanITcare app (35).

Utilizing the sensors embedded in smart mobile phones, a new generation of well-being applications have been designed to automatically monitor multiple aspects of physical and mental health, for example, changes in the speech production process, which happens during stress. Therefore, microphones, embedded in mobile phones and carried ubiquitously by people, provide the opportunity for the non-invasive and continuous detection of voice-based stress (75). StressSense (75) and BeWell+ (27) are sensor-enabled smartphone apps designed to monitor user behavior. StressSense is designed to identify stress from the human voice using microphones embedded in mobile phones. BeWell+ continuously monitors user behavior, namely sleep, physical activity, and social interaction, and promotes improved behavioral patterns via feedback rendered as notifications, and this has been shown to successfully convey information and increase awareness (31). Ultimately, in the study by Boukhechba et al. it is suggested that mobility features, such as location entropy, are negatively associated with social anxiety with socially anxious students avoiding public areas and engagement in leisure activities. These findings were demonstrated by passively generating GPS data from an app installed on participants' personal mobile phones. Last, Ramon-Fernandez et al. propose an environmental stressor monitoring system to identify stressful environments based on parameters such as level of noise in the room, flow of people moving through it, temperature, luminosity, and air quality (46).

### Depression

Depression is one of the most common mood disorders, and it is characterized by the absence of a positive affect; loss of interest in activities and experiences; low mood; and a range of associated emotional, cognitive, physical, and behavioral symptoms (94). Interpretation of data from smartphone-based geolocation sensors has identified the association between digital markers and mental illness concepts, especially mood (97). These geolocation-derived digital markers include number of locations visited, distance traveled, and time spent at a specific location. GPS embedded in participants' smartphone, GSM cellular network, and Wi-Fi are the common tools for measuring geolocation (97). Conversation frequency and duration, sleep disruption, social withdrawal and avoidance, mobility and GPS features are among the factors related to depression that can be detected using smartphone sensors (3). The evidence-based behavior (eB2) app has been designed to identify changes in the mobility patterns of patients with depression based on the smartphone's native sensors and advanced machine learning and signal processing techniques. The app captures inertial sensors, physical activity, phone calls and message logs, app usage, nearby Bluetooth and Wi-Fi connections, and location. The study has proposed that some specific mobility pattern changes can be indicators of relapses or clinical changes; however, that might not always be the case (30). Similarly, in a study by Ben-Zeev et al. it is suggested that increased geospatial activity is associated with better depression scores. Nevertheless, the same study identified that geospatial activity and sleep duration are inversely associated with daily stress, and no correlations were identified between sleep duration, geospatial activity, or speech duration and loneliness (33). Furthermore, a momentary assessment study aims to predict future depressed mood within hour-to-hour time windows. Utilizing ideographically weighted machine learning models and passive mobile phone sensor data, the study shows the significant correlation between observed and predicted hourly mood (34).

A study by Jin et al. proposes an attention-based deep learning architecture combined with wearable sensing devices, which can effectively classify mental states by analyzing behavior signals and speech under different emotions (17). The sensing system of the wearable device is used for analyzing the degree of anxiety and depression and consists of 6-D acceleration and angular sensors, a temperature and humidity sensor, MEMS microphones, and an audio code unit (17). Also, a study by McGinnis et al. sought to identify anxiety and depression in children under the age of eight by developing objective measures. To recognize children with internalizing disorders, the study utilizes an instrumented fear induction task and captures the six degree–of-freedom movement of a child using data from a belt-worn inertial measurement unit. The results suggest that the collected motion data are sensitive to behaviors that are representative of child psychopathology (24). Last, a fingertip PPG sensor has been developed by Dagdanpurev et al. as a major depressive disorder (MDD) screening system (19). The system employs autonomic nerve transient responses induced by a mental task and logistic regression analysis to identify MDD patients from healthy subjects. The self-monitoring system achieved 83% sensitivity and 93% specificity in MDD screening determined by ECG-derived HRV (20).

### Bipolar Disorder

**Figure 8:**
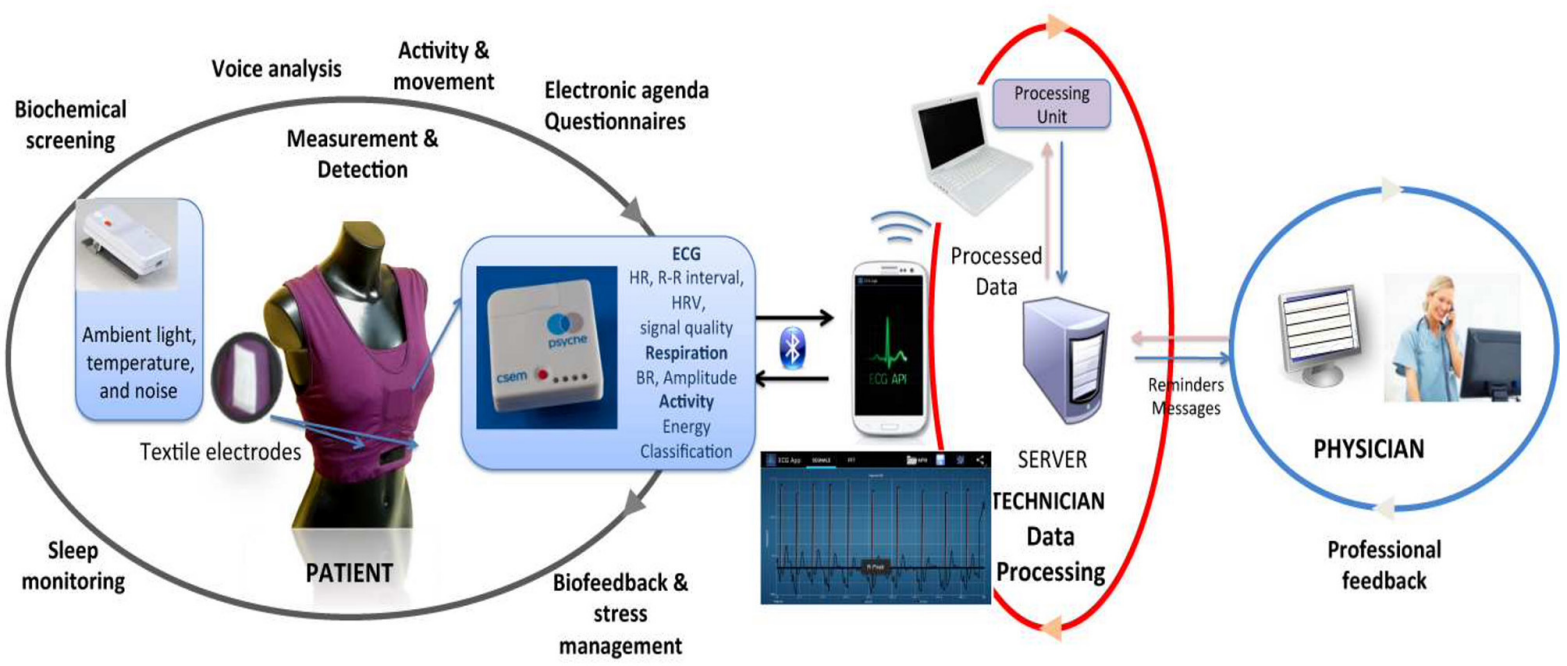
Overview of the PSYCHE wearable monitoring system. The system involves a T-shirt with embedded textile sensors, mobile application for physiological signal acquisition from the wearable platforms, and feedback to patient and clinician [Modified from Lanata et al., *IEEE Journal of Biomedical and Health Informatics*, 2015 (15)].

Bipolar disorder (BD), characterized by recurrent episodes of depressed and manic mood states, is a leading cause of disability worldwide and is associated with significant functional impairment (98). In the management of BD, PSYCHE, a personalized wearable monitoring system was trialed on 10 bipolar patients exhibiting depression, hypomania, mixed state, and euthymia symptoms to assess the pathological mood states by recording physiological signals (Figure 8). The pathological mood states were assessed using a data mining strategy by recording the physiological signals through information collected from the autonomic nervous system. PSYCHE employs a commonly used measure of entropy to analyze more than 400 h of cardiovascular dynamics and characterize the symptoms. This helped to improve the diagnosis and management of psychiatric disorders (15). The system consists of a comfortable t-shirt with embedded sensors, such as textile electrodes, to monitor electrocardiogram (ECG) for HRV series, piezoresistive sensors for respiration activity, and tri-axial accelerometers for activity recognition, and provides a smartphone-based interactive platform and data visualization to the patient and physician, respectively. The t-shirt with the above-described sensors is coupled with embedded electronics to acquire and store the data and an internal tri-axial accelerometer to monitor movement activity (15). Once the electronic device is detected by a mobile application through Bluetooth, the physiological data streaming is initiated. Several algorithms are then used to process the physiological data collected and correlate it with the mental health status of the patient. The data is then uploaded automatically by patients who are connected at home to produce predictive results that allow the physician to optimize the patient's treatment. Therefore, the textile-integrated platform together with the smartphone framework offered long-term acquisition of HRV data and helped discriminate different pathological mood states and investigated the response to treatment on BD patients (15). It has been demonstrated that data coming from the PSYCHE platform (long-term HRV series) could be considered as a viable biomarker for discriminating of bipolar patients and their response to treatment (15).

In a study by Prociow et al. wearable sensing techniques were coupled with environmental sensors to observe the areas of life influenced by BD and match the collected information with bipolar symptoms (16). The system included a wearable light sensor and a belt-worn accelerometer, which was used to detect restless behaviors, such as psychomotor agitation and increased or decreased physical activity to identify mood states. Moreover, the environmental system included bed sensors and light detectors installed in the patients' home to monitor altered sleep patterns, such as insomnia, hypersomnia, and self-deprivation of sleep, which are important diagnostic indictors that a manic or depressive episode is occurring. Basic processing performed on the behavioral data yielded from these sensors provided information about early effects of a bipolar episode on activity patterns. The study was performed on four healthy subjects and one participant with BD. The recruited bipolar patient remained euthymic (asymptomatic) throughout the monitoring period (16).

**Figure 9:**
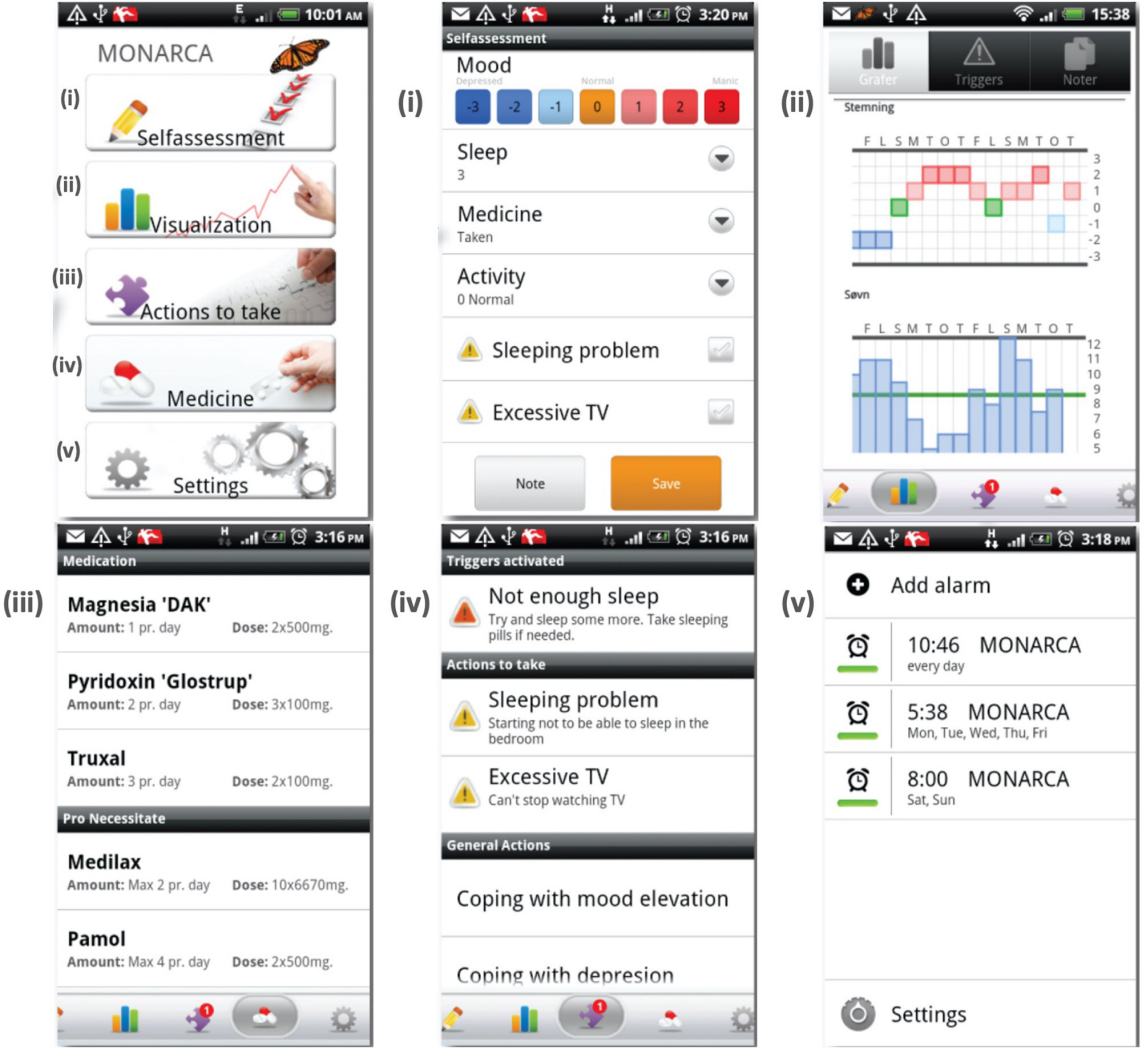
The MONARCA application user interface [Modified from Bardram et al., 2013 (99)].

Combining self-reports and passive sensor data, several studies have sought to monitor mood fluctuations involved in bipolar disorder. MoodRhythm is a smartphone application that combines self-report and passive sensing via the use of smartphone sensors for the long-term monitoring of bipolar disorder. The application incorporates existing self-report strategies from interpersonal and social rhythm therapy and combines them with inputs from smartphone sensors (28). Additionally, the MONARCA system was developed to investigate the monitoring, treatment and prediction of bipolar disorder episodes (Figure 9) (67, 78, 100–102). The MONARCA project is a smartphone-based behavior monitoring technology that leverages a variety of phone sensors to detect changes in mental states. The system also includes a feedback loop between patients and clinicians through a web portal that provides detailed historical overviews of a patient's data to generate measures of illness activity (29, 55). The system investigates self-monitored (subjective) or sensor-based automatically generated (objective) behavioral data to monitor mood states in patients with bipolar disorder. The automatically generated behavioral data is collected by smartphones supporting different types of sensors. Some of the sensor-based data on measures of illness activity include phone usage, social activity measured as the number and length of incoming and outgoing phone calls and text messages, physical activity measured through a step counter, mobility based on location estimation, and speech activity collected by extraction of different voice features without recording the actual conversation. The study was performed for a 9-month trial period to investigate the correlations between subjective and objective behavioral data and the severity of depressive and manic symptoms. Altogether, the MONARCA system has been shown to recognize the clinical states of bipolar patients (depression/mania) with 72–81% accuracy based on location features. The system also shows high acceptance within the patients and health care providers and is proved to be effective in reducing the level of bipolar symptoms (29). Among other studies targeting bipolar patients, Social Information Monitoring for Patients with Bipolar Affective Disorder (SIMBA) is a smartphone monitoring app tracking daily mood, physical activity, and social communication in bipolar patients (32). Using the data collected from the SIMBA app, it has been suggested that the distance traveled by the bipolar patient is negatively associated with manic states (32). However, this is contrary to the findings of a study by Faurholt-Jepsen et al. (67) suggesting that the number of changes in communication are positively associated with manic states.

### PTSD

PTSD follows significant trauma and is characterized by emotional numbness; intrusive reliving of the traumatic episode; disturbed sleep, including nightmares; and hyperarousal, such as exaggerated startle responses (94). A couple of wearable monitoring systems have been developed for individuals with PTSD. Namely, a wrist-worn actigraphy sensor has been investigated for the monitoring of activity, sleep, and circadian patterns in PTSD, which involves long-lasting symptoms, such as avoidance behaviors and sleep disturbance. The proposed sleep detection algorithm has been demonstrated to match the participants' sleep diaries. Also, determined from the greater nocturnal activity and awakenings, participants with PTSD were found to exhibit more fragmented sleep patterns compared with non-traumatized control groups (22). Furthermore, a wearable sensor system has been designed and implemented consisting of a neoprene band worn on the ankle as well as an optional custom-designed ECG heart monitor worn on the chest, both containing a Bluetooth radio that enables communication with the patient's mobile phone. The sensor bands contain circuitry for measuring EDA, three-axis motion, temperature, and ECG. The system has been studied for PTSD and drug addiction and works by presenting therapeutic and empathetic messages to the patient in the tradition of cognitive behavioral therapy when a specific arousal event is detected (13).

### Schizophrenia

Schizophrenia is a neuropsychiatric syndrome exhibiting psychotic symptoms, such as hallucinations and delusions; negative symptoms, including loss of motivation and blunted affect; and last, cognitive symptoms, including impairments in attention, working memory, verbal fluency, and various aspects of social cognition (94). Continuous remote monitoring and identification of subjective and objective indicators of psychotic relapse is known to improve the management of schizophrenia-spectrum disorders. Accordingly, the CrossCheck project has identified statistically significant associations between passive smartphone sensor data and self-reported indicators of mental health in schizophrenia (31, 90). The study suggests that lower rates of physical activity are linked to negative mental health. Also, it is demonstrated that patients showing lower sociability indicated particularly with fewer conversations during the morning and afternoon periods are likely to exhibit negative feelings. Nevertheless, it has been indicated that an increased number of phone calls and SMS messages can also be associated with negative dimensions of mental health as some individuals prefer to use the phone instead of face-to-face communication during negative mental states. Among other findings from monitoring of individuals with schizophrenia-spectrum disorders is that visiting fewer new places and going to bed later is associated with negative feelings, and getting up earlier is suggested to be linked with positive mood (103, 104).

## Discussion

The current review presents a comprehensive analysis of developed sensor-based strategies in the management of mental disorders that seek to monitor physiological and behavioral data as indirect measures of health states. The literature review identifies studies investigating monitoring systems for the management of different mental disorders, including stress and anxiety disorders, depression, BD, cognitive impairment, PTSD, and schizophrenia. Principally, mental health monitoring systems collect and interpret physiological and behavioral data to relate them to the symptoms of mental conditions and achieve digital phenotyping. Thereby, a number of environmental, wearable, and mobile phone sensors have been employed to monitor parameters such as HRV, ST, EMG, BVP, blood pressure, etc. In each study, the type and variety of sensors were determined based on the application of the monitoring system as well as the behavioral and physiological parameters that were expected to be altered as a consequence of a specific disorder. The predicted mood states were often compared with the self-reports coupled with the monitoring systems. The majority of the studies have also suggested specific correlations between the collected behavioral and physiological parameters and the studied mental disorder. However, it should be noted that most of the developed technologies have not been thoroughly validated for their usefulness in clinical applications. Furthermore, the majority of monitoring systems target the general public and healthy individuals for health and fitness applications and are not certified as medical devices, and their impact on emotional states needs to be assessed (58). Also, these studies are subject to small sample sizes and short follow-up times. Therefore, there remain many technical and practical issues to overcome in digital approaches to mental disorders that include maintaining compliance over time, constraints on battery, manufacturing, durability, integration, constraints with analysis and interpretation of information, data accuracy, ethics/privacy issues, acceptability, and engagement issues (98). The mental health monitoring systems employing user-independent (general) models might also need to consider diversities such as age and ethnicity when introducing a predictive model. Moreover, despite the rapid development of sensing technology for monitoring physiological and behavioral data, challenges remain for the effective use of this abundant data. These challenges include processing the raw sensor data and managing the noise and artifacts, considering intrapersonal and interpersonal differences when interpreting the data, cost-effectiveness, and practicality of the device, providing privacy-protecting strategies, designing an accessible data storage and monitoring platform, and providing personalized coaching strategies. The only way to overcome these challenges is by interdisciplinary teams sharing expertise and methods and involvement of end users.

Although body-worn sensors that recognize human activities have the advantage of being at the user's hand, deploying these systems imposes some constraints, including protecting the user's privacy, being lightweight, offering limited computing power, handling the real world's noisy data and complexities, and comprising machine-learning algorithms that are trainable without requiring extensive human supervision. For instance, to avoid privacy issues, speech information should be automatically evaluated without preserving raw audio data (5). Another constraint is that, among wearable systems, the participant acceptability varies between different device forms, which is often related to the appearance and comfort of the wearing sensors. Accordingly, in a study by Huberty et al., it is demonstrated that wrist and upper arm sensors are more preferred than a sensor worn on the non-dominant hip in a sample of middle-aged women (105). Moreover, the results from a patient trial address the issue of non-adhering due to the discomfort of carrying extra devices, forgetfulness, and lack of familiarity with personal technology (16). Therefore, wearing and carrying extra devices on a day-to-day basis has been found to be impractical, and pursuing the environmental sensing via only a mobile phone seems to be ideal. However, it should be noted that wearable sensor features including skin conductance and temperature were found to be more accurate (78.3% accuracy) in identifying stress and poor mental health than mobile phone features (73.5% accuracy) such as phone usage and mobility patterns (106). Another shortcoming of mobile phone apps monitoring behavioral markers is their noticeable effect on the battery life, which can be a problem for users (61). Altogether, incorporating sensors monitoring physiological and behavioral parameters into a single, easy-to-use device has potential for the development of a practical mental health monitoring system. Furthermore, there is a crucial need for transparency and collaborative partnership between providers and patients because trust in the organization that collects the data and the purpose of data collection are the main factors for acceptability of the sensing systems. Additionally, ambient sensors can be expensive and limited to a particular physical space, their proper functioning requires calibration, and they might also raise privacy concerns. Nevertheless, these sensors have the advantage of not requiring direct contact with the user, hence, being free of wearing or carrying devices. Last, there is crucial need for patients, psychiatrists, and psychologists to adopt such means into their treatment and general practice (16).

When considering physiological and behavioral data as measures of mental health, it is important to consider that sensors do not sense the mental state itself, but a behavior that is the consequence of an underlying physiological alteration. Furthermore, relying solely on physiological sensor data might not be optimal as body changes may occur from other factors, such as food or medication intake and physical exercise. Thus, the mental health monitoring system should also consider the individual characteristics of each user. Another important aspect that should be considered is that the nature of the collected data from these sensors differs from the usual information that is available to and discussed with health care professionals. Patients have been found to express more willingness to consent to passive data retrieval from less personal sources, such as mobile phone screen time, whereas respondents were found to be at least willing to consent to more personal data, such as communication and location data, as well as audio recording and analysis (107). It has been noted that participants' comfort with sharing data depends on the data type and the recipient. Although some individuals were shown to be comfortable with sharing their health information, including sleep, mood, and physical activity with their doctors, they were less comfortable sharing personal data, such as communication logs, location, and social activity, especially with the electronic health record systems and their family (108).

Although there have been great advances in the automatic monitoring of mental health, there remain challenges that can pose several research opportunities. One of the key steps in the development of mental health monitoring systems is data labeling, which is used for finding associations between the sensor data and the corresponding true mental state at that time span. Therefore, machine learning models should be further explored to evaluate their potential for mental health monitoring. Another aspect that needs to be considered when monitoring physiological and behavioral patterns is the inter-user differences. As aforementioned, user-dependent models are suggested to perform better than user-independent (general) models, and hybrid models can be used to combine the strengths of both models (68, 75). Therefore, the potential of this type of hybrid model should be further explored. Additionally, intra-user differences that refer to the variability of physiological and behavioral patterns for the same user should also be addressed. Last, the mental health monitoring systems need to be clinically validated and should be integrated with other systems, including user databases, administrative tools for caregivers and physicians, and support systems to achieve optimum communication platforms. The application of multimodal sensing technologies along with the appropriate machine learning methods is of significance for achieving monitoring of mental health.

## Conclusion

In conclusion, facile physiological and behavioral sensing systems have the potential to directly enhance the management and monitoring of mental health. Use of these devices can increase patients' self-awareness, which can positively affect the management of their condition. This consciousness about one's mental state can prevent the worsening and further adverse effects of many mental disorders. Therefore, technology needs to be established in mental health as it has in other fields of medicine to provide practical and point-of-care devices for those who are affected. Development of monitoring devices will aid patients in the management of their condition and immensely increase their quality of life.

## Author's Note

The review paper has been written by MS under the supervision of MQ and PK. MQ and PK have reviewed the paper manuscript and made comments and statements. The authors of the review have complementary expertise, ensuring a balanced viewpoint. PK is a leader in the field of medical devices, developing innovative applications for the technology in a range of settings. MQ is a lecturer in biomedical engineering with expertise in optical spectroscopy and developing minimally-invasive monitoring devices. MS research focuses on non-invasive lithium monitoring in the management of bipolar disorder. Together, the authors provide a broad perspective on the topic, ensuring that the review is of interest to a wide audience.

## Author Contributions

MS and MQ: conceptualization and methodology. MQ and PK: validation, investigation, and supervision. MS: writing-original draft preparation, writing-review, and editing. All authors listed have made a substantial, direct and intellectual contribution to the work, and approved it for publication, have read and agreed to the published version of the manuscript.

## Conflict of Interest

The authors declare that the research was conducted in the absence of any commercial or financial relationships that could be construed as a potential conflict of interest.
